# Rift Valley Fever Virus Exposure in Camels and Horses Across Northern Nigeria Livestock Markets

**DOI:** 10.3390/pathogens15030258

**Published:** 2026-02-28

**Authors:** David Odion Ehizibolo, Olumuyiwa Oyekan, Nicodemus Mkpuma, Habibu Haliru, Ibrahim Garba, Isa Zayyad Turaki, Elizabeth Ene Williams, Agom Danmarwa, Abdullahi Mohammed, Musa Abdullahi Muhammad, Mansur Abubakar, Corrie Brown, Bonto Faburay

**Affiliations:** 1 National Veterinary Research Institute, Vom 930001, Plateau State, Nigeria; 2 Veterinary Department, Ministry of Agriculture and Natural Resources, Dutse 720101, Jigawa State, Nigeria; 3 Department of Veterinary Services, Ministry of Animal Health and Fisheries, Sokoto 840103, Sokoto State, Nigeria; 4 LifeStock International, Athens, GA 30606, USA; 5 Foreign Animal Disease Diagnostic Laboratory, National Veterinary Services Laboratories, National Bio and Agro-Defense Facility, United State Department of Agriculture, Manhattan, KS 66505, USA

**Keywords:** Rift Valley fever virus, seroprevalence, camel, horse, livestock market, Nigeria

## Abstract

Rift Valley Fever (RVF) is a neglected vector-borne zoonotic disease of significant veterinary and public health concern in Sub-Saharan Africa. This study investigated the seroprevalence of Rift Valley Fever Virus (RVFV) exposure and associated risk factors among camels and horses marketed in northern Nigeria. A total of 1117 animals were sampled, comprising camels (812) and horses (305), across three major livestock markets (Maigatari, Maiduguri, and Illela). The overall seroprevalence was 18.8% (95% CI: 16.6–21.2%), with a striking six-fold disparity: camels showed a prevalence of 24.4% (95% CI: 21.6–27.4%), while horses exhibited only 3.9% (95% CI: 2.1–7.0%). Significant geographic clustering was observed, with Illela camels recording the highest prevalence (34.8%) compared to those in Maigatari (20.3%) and Maiduguri (20.2%). There were no significant associations with age or sex among camels. However, in horses, females were significantly more likely to test positive than males (OR = 0.27, 95% CI: 0.07–0.97). These findings demonstrate endemic RVFV circulation in Nigerian livestock, highlighting species- and location-specific differences, and underscore the zoonotic risks within regional and transboundary livestock trade networks.

## 1. Introduction

Rift Valley Fever (RVF) is an acute viral disease that affects both domestic and wild animals as well as humans [1]. The causative agent, Rift Valley Fever Virus (RVFV), is an RNA virus belonging to the genus *Phlebovirus* within the family *Phenuiviridae* [2]. Transmission of RVFV occurs mainly through bites of infected mosquitoes, particularly *Aedes* and *Culex* species, and other haematophagous arthropods. Human infections may also arise through direct contact with the blood, tissues, or body fluids of infected animals, representing an important occupational hazard for farmers, veterinarians, and abattoir workers [3]. In addition, animal products such as fresh or frozen meat, milk, wool, bones, hides, and skins have been implicated as potential sources of infection [4]. The virus was isolated from milk and manure of infected animals during the 1977 outbreak in Egypt, suggesting that consumption of contaminated raw milk might constitute a route of human infections [5]. Furthermore, the long-distance movement of viremic animals along trade and transhumance routes has been reported to facilitate the spread of RVFV across regions [6].

The epidemiology of RVF is complex, involving multiple hosts and vectors including mosquitoes, wild animals, domestic livestock, and humans [7]. Among livestock, cattle, sheep, goats, and camels are particularly susceptible and serve as amplifying hosts, thereby enhancing viral circulation within endemic areas [8]. Outbreaks of RVF are often associated with periods of persistent heavy rainfall that favor mosquito breeding and proliferation. During inter-epidemic periods, the virus is maintained at low levels in enzootic cycles involving mosquitoes and susceptible vertebrate hosts [9].

Rift Valley Fever remains endemic across sub-Saharan Africa, with numerous outbreaks recorded in countries such as Kenya, Uganda, Tanzania, South Africa, Sudan, Senegal, Mauritania, and Niger, events that have resulted in considerable socio-economic losses among pastoral and agro-pastoral communities [10,11,12,13,14,15]. Although historically restricted to Africa, the geographic range of the virus has expanded, with outbreaks reported on the Arabian Peninsula, highlighting its transboundary transmission potential [16]. In Nigeria, no confirmed clinical cases of RVF have been reported in indigenous livestock; however, a fatal infection was documented in a Merino sheep imported from South Africa [17], and serological evidence of RVFV exposure has been reported in humans, wildlife, and various domestic animal species [18,19,20,21,22,23,24,25,26,27]. More recently, molecular detection of RVFV in arthropod vectors has further confirmed the presence of the virus within the country [28]. Serological evidence of RVFV exposure in camels has been recorded across several regions of Nigeria [22,29,30]; however, to date, only a single study has reported evidence of RVFV exposure in horses in the country using the haemagglutination-inhibition test [22]. The majority of camels originating from eastern and northern Africa are traded across multiple borders before arriving in northern Nigeria, a movement pathway that may facilitate transboundary viral spread [31]. Collectively, these reports highlight the continued circulation of RVFV in Nigeria and reinforce the need for ongoing surveillance, particularly in livestock that may function as key sentinels.

Nevertheless, limited information exists regarding the occurrence of RVFV in camels and horses in Nigeria. In light of these gaps, the present study was designed to determine the seroprevalence of RVFV in camels and horses in northern Nigeria and to explore their potential role in the epidemiology and transmission dynamics of the disease.

## 2. Materials and Methods

### 2.1. Study Area and Animals

**Figure 1:**
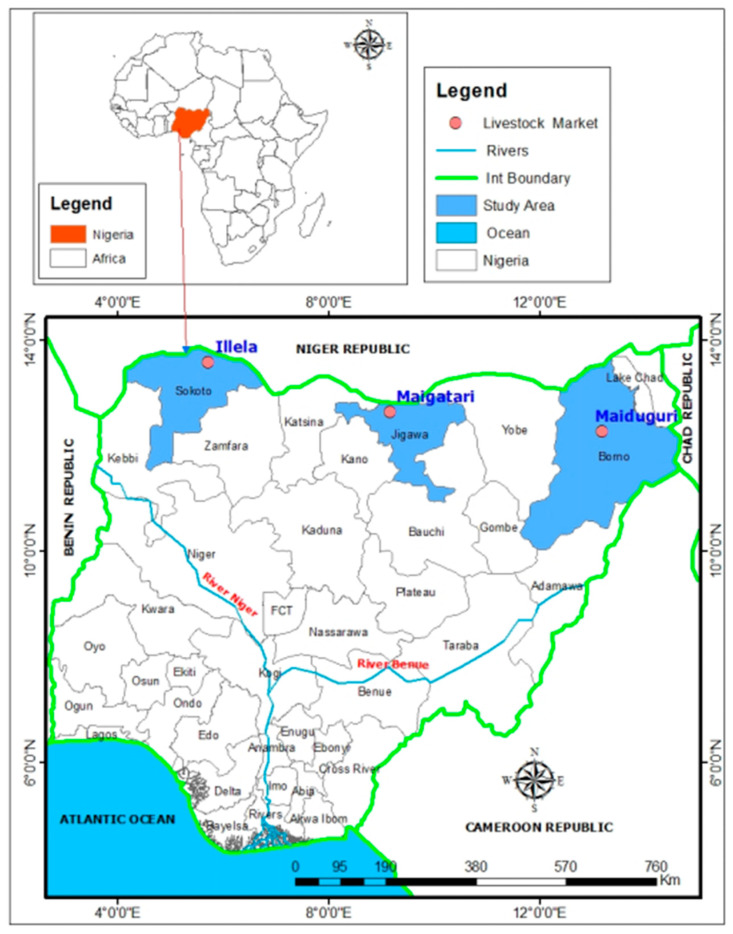
Map of Nigeria showing study locations.

This study was conducted in major livestock markets across three northern Nigerian states: Maiduguri market in Borno State, Maigatari market in Jigawa State, and Illela market in Sokoto State. All three markets are located near international borders with Niger Republic and the Republic of Chad (Figure 1). From June 2023 to July 2024, a cross-sectional active surveillance system was implemented in these high-throughput markets, which receive weekly shipments of livestock, including animals originating from neighboring countries. Horses were sampled exclusively at Maigatari livestock market (*n* = 305), as they were not traded in the other markets. Camels were sampled across all three markets: Maigatari (*n* = 257), Illela (*n* = 230), and Maiduguri (*n* = 325). Each market was visited weekly, over approximately 45–47 visits, and on each weekly visit to a market, a convenience sample of five animals per species was selected. Animals were selected using convenience sampling due to logistical constraints in livestock market settings. All sampling procedures were conducted with the consent of animal owners or handlers.

### 2.2. Data and Sample Collection

Data were collected at the animal level, and information obtained included estimated age, sex, and geographic origin. Age classification was based on a combination of dentition assessment (tooth eruption and wear patterns) [32,33] and owner-reported age. Animals were categorized as young (≤4 years) or adult (>4 years). Whole blood samples were collected from the jugular vein using sterile syringes and needles and transferred into serum separation tubes. After allowing blood to clot at ambient temperature (20–25 °C) for 30 min to 2 h, samples were stored in cool boxes with ice packs and transported to the laboratory for same-day processing. Serum was aliquot into clearly labeled sterile cryovials and stored at −20 °C until analysis.

### 2.3. Detection of Anti-RVFV Antibodies

A competitive enzyme-linked immunosorbent assay (cELISA) was used to detect antibodies indicating previous exposure to Rift Valley fever virus (RVFV). Serum samples were analyzed using the multi-species ID Screen^®^ RVF IgG ELISA kit (ID-Vet Innovative Diagnostics, Grabels, France) according to the manufacturer’s instructions. Assay validity was confirmed when the mean optical density (OD) of the positive control (OD_450_ PC^+^) was less than 0.3 times that of the negative control (OD_450_ NC), and the mean OD of the negative control exceeded 0.7. All sera and controls were tested at dilutions of 1:1 and optical densities were read using a spectrophotometer Multiskan^®^ ELISA reader (Thermo Scientific, Waltham, MA, USA), and the OD was determined at 450 nm. For each sample, the competition percentage (S/N %) was calculated as: S/N % = (OD_450_ Sample/OD_450_ NC) × 100. Samples with S/N % ≤ 40% were considered positive, those between 40–50% were classified as doubtful (inconclusive), and those with S/N % > 50% were regarded as negative.

### 2.4. Data Analysis

Data were analyzed using R statistical software (version 4.3.0). Descriptive statistics were computed for all variables, with categorical data summarized as frequencies and percentages. Seroprevalence estimates were calculated with 95% confidence intervals (CIs) using test of binomial proportions. Differences in seroprevalence between species and across locations were assessed using chi-square tests, and a significance level of 0.05 was used. Risk factor analyses were conducted separately for camels and horses because of differences in species distribution across markets and marked disparities in seroprevalence. Univariable logistic regression was used to evaluate associations between RVFV seropositivity (positive vs. negative) as the binary outcome variable. Explanatory variables, including age category (young as reference) and sex (female as reference). Odds ratios (ORs) with 95% CIs were estimated to quantify the relative odds of RVFV seropositivity compared with the specified reference categories. Multivariable models were not fitted because species-specific differences in outcome frequency, particularly the low number of seropositive horses, precluded stable multivariable estimation and meaningful cross-species comparison.

## 3. Results

### 3.1. Descriptive Characteristics of Livestock Sampled Across the Study Markets

A total of 1117 animals were sampled across the three livestock markets (Table 1), comprising 812 camels (72.7%) and 305 horses (27.3%). Camels were sampled across all three study locations, with the highest number obtained from Maigatari market (325; 29.1%), followed by Maiduguri (257; 23.0%) and Illela (230; 20.6%). In contrast, all horses were sampled exclusively at Maigatari market. Overall, 601 animals (53.8%) were classified as young (≤4 years), while 516 (46.2%) were adults (>4 years). Among camels, 480 were young and 332 were adults, whereas among horses, 121 were young and 184 were adults. The sex distribution was relatively balanced, with 587 males (52.6%) and 530 females (47.4%). Specifically, camels comprised 419 males and 393 females, while horses included 168 males and 137 females.

### 3.2. Species-Specific and Spatial Variation of RVFV Seroprevalence

Overall, RVFV seroprevalence among the sampled livestock was 18.8% (95% CI: 16.6–21.2). The most striking finding is the profound interspecies variation in RVFV seroprevalence, with camels demonstrating a 24.4% overall seroprevalence compared to only 3.9% in horses (Table 2) representing a six-fold difference in species-specific seroprevalence. The 95% confidence intervals for these species-specific prevalence rates do not overlap (camels: 21.6–27.4%; horses: 2.1–7.0%), indicating that this difference is statistically significant (*p* < 0.05). In Maigatari livestock market, the only location where both camels and horses were sampled, camels showed a higher RVFV seropositivity than horses (20.3% vs. 3.9%). RVFV seroprevalence across livestock markets is also shown in Table 2. Among camels, substantial variation in seroprevalence was observed between markets, with Illela demonstrating the highest antibody prevalence at 34.8% (95% CI: 28.7–41.4%), significantly (*p* < 0.05) exceeding the rates observed in Maigatari (20.3%; 95% CI: 16.2–25.2%) and Maiduguri (20.2%; 95% CI: 15.6–25.8%).

### 3.3. Risk Factors Associated with RVFV Seropositive Camels

Among the 812 camels examined, no significant associations were found with RVFV exposure status (Table 3). Although age stratification revealed a modest numerical difference, with young camels exhibiting a higher prevalence (26.2%; 95% CI: 22.4–30.4%) than adults (21.7%; 95% CI: 17.4–26.5%)—the odds ratio of 1.29 (95% CI: 0.92–1.79. *p* = 0.137) indicates that this difference is not statistically significant, as the confidence interval includes unity. Similarly, sex-based analysis did not identify a statistically significant associations with RVF seropositivity among camels. Males had slightly lower odds of being seropositive compared with females (OR = 0.85, 95% CI: 0.62–1.17, *p* = 0.313), indicating a 15% lower likelihood, although this difference was not significant.

### 3.4. Risk Factors Associated with RVFV Seropositive Horses

The horse cohort (*n* = 305) exhibited markedly different risk factor patterns (Table 4); however, interpretation is limited by the small number of RVFV-seropositive animals (*n* = 12). Age-related analysis showed that adult horses had nearly twice the seroprevalence of young horses (4.9% vs. 2.5%), corresponding to an odds ratio of 2.02 (95% CI: 0.54–7.63, *p* = 0.293). The most notable finding emerged from the sex-based assessment. Female horses exhibited substantially higher prevalence (6.6%; 95% CI: 3.0–12.1%) compared with males (1.8%; 95% CI: 0.4–5.1%). The odds ratio for males (0.27; 95% CI: 0.07–0.97, *p* = 0.046) was the only association reaching statistical significance across both species; however, this finding should be interpreted cautiously given the small number of seropositive horses and wide confidence interval. Male horses showed approximately 73% lower odds of being seropositive compared to females.

## 4. Discussion

This study provides clear serological evidence of prior exposure to RVFV in northern Nigeria, with an overall seroprevalence of 18.8% (95% CI: 16.6–21.2%) among camels and horses sampled from international livestock markets. These markets function as major nodes for transboundary animal movement across West Africa, and the observed seroprevalence reflects cumulative exposure of highly mobile livestock populations. Camels exhibited a markedly higher seroprevalence than horses, reinforcing their greater likelihood of RVFV exposure over time. Among camels, apparent differences in seroprevalence were observed across markets, with the highest levels recorded among animals sampled at Illela. Similar to findings reported by Oragwa et al. [26]. This pattern likely reflects the geographic origins of the animals brought to the market, rather than exposure occurring at the market itself, highlighting the influence of source populations on observed RVFV seropositivity. Such clustering is plausibly driven by intense cross-border livestock movements involving Niger and Burkina Faso, as well as by ecological and environmental conditions that may favor RVFV transmission along transboundary trade routes. Transboundary movement of livestock into northern Nigeria is well documented [18,34], and RVF outbreaks have occurred in neighbouring Niger Republic [35]. The seroprevalence observed among camels aligns with more recent reports from Nigeria and other African countries, where values ranging from 19.9% to 36.5% have been documented [30,36,37,38]. In contrast, the prevalence reported here is considerably higher than earlier Nigerian estimates of 3.3% and 3.13% [22,29], possibly reflecting changes over time in surveillance sensitivity, study design, geographic coverage, or cumulative exposure resulting from increased livestock movement. Although camels are often described as potential amplifying hosts during inter-epidemic periods [39], the present findings should be interpreted cautiously. The detected antibodies indicate past exposure and do not provide evidence of active infection or ongoing RVFV circulation at the time of sampling. Nonetheless, the high seroprevalence observed, particularly among camels in transboundary markets, underscores their value as sentinels for monitoring potential risk for RVFV exposure and informing risk-based surveillance in northern Nigeria and the wider West African region. Across West and Central Africa, information on RVFV infection in equids remains sparse, as surveillance has historically prioritized ruminant species. Additionally, it is unclear if horses play any role in the epidemiology of RVF. In the present study, RVFV seroprevalence in horses was low (3.9%), supporting the view that equids experience limited exposure to the virus in this region. This estimate is lower than the 9.8% previously reported in Nigeria by Olaleye et al. [22]; however, that earlier finding was based on a small sample size (5 seropositive animals out of 51 horses), which might have led to an overestimated true prevalence. Variations in seroprevalence between studies may reflect differences in ecological settings, vector abundance, or management and husbandry practices that influence mosquito exposure, although these factors were not directly assessed in the present study. Our findings are further supported by a recent investigation in Bauchi State, Northeast Nigeria, where no RVFV antibodies were detected among 200 horses, despite evidence of RVFV exposure in cattle and wildlife sampled from the same area [25]. Taken together, these data consistently indicate that horses in northern Nigeria and surrounding regions exhibit low RVFV seroprevalence and are likely less involved in RVFV transmission dynamics compared with camels and other domestic livestock species.

In East Africa, major RVF epidemics have been documented in countries such as Kenya, Tanzania, and Somalia, often occurring after periods of unusually heavy rainfall that promote large-scale mosquito amplification [40]. During such outbreaks, seroprevalence levels exceeding 40% have been reported, which contrasts sharply with the lower seroprevalence observed in Nigeria and other parts of West Africa. This disparity suggests that, unlike the explosive epidemic patterns seen in East Africa, RVFV transmission in West Africa is more likely characterized by persistent, low-intensity exposure. Comparisons between the two regions should, however, be made cautiously, as heavy rainfall events that drive large RVF outbreaks are relatively uncommon in West Africa. These differences likely reflect regional ecological and climatic variations that influence vector abundance, species composition, and transmission dynamics. Nevertheless, the extensive transboundary livestock trade within West Africa may facilitate silent, cross-border dissemination of RVFV, contributing to sustained low-level exposure across the region.

Risk factor analyses in this study revealed distinct epidemiological patterns between camels and horses, alongside limitations related to statistical power, particularly for equids. Among camels, no significant associations were observed between RVFV seropositivity and demographic variables such as age or sex. Although young camels exhibited a slightly higher prevalence than adults, this difference was not statistically significant, suggesting relatively uniform exposure across age and sex groups. This finding is consistent with earlier study in Nigeria, Musa et al. [30], who also reported no sex-based differences, but observed a significant age-related association, with older animals exhibiting higher RVFV antibody prevalence. A similar age-related association was reported in studies from from Kenya and Sudan [39,41]. While seroprevalence for many infections typically increases with age due to cumulative exposure, similar patterns of higher or comparable RVFV seroprevalence in younger animals have been reported. For example, in a study of cattle in the Democratic Republic of the Congo, younger cattle exhibited a higher RVFV seroprevalence than older animals, although the difference was not statistically significant [42], suggesting recent or ongoing transmission can influence age–seroprevalence relationships rather than cumulative lifetime risk alone. In the present study, the observed age-related trend was modest and not significant, indicating that RVFV exposure was relatively uniform across age categories and should be interpreted with caution.

In horses, higher seropositivity was observed among females and adults; however, interpretation is constrained by the small number of seropositive cases and wide confidence intervals. While the higher odds of seropositivity among adult horses may suggest cumulative exposure over time, this pattern should be interpreted cautiously. Across much of Africa, female livestock are typically retained longer for breeding and productive purposes, whereas males are fewer and often slaughtered earlier, potentially skewing observed age and sex distributions and influencing apparent risk patterns.

The detection of RVFV antibodies in both camels and horses provides further evidence of prior exposure in these species and suggests involvement in the broader RVFV transmission ecology. Given the zoonotic nature of RVF, these findings have important public health implications. Individuals engaged in livestock husbandry, trade, slaughter, and veterinary practice may face increased occupational risk through contact with infected tissues or body fluids. Strengthening integrated animal–human surveillance systems, enhancing vector control strategies, and fostering intersectoral collaboration are therefore critical to mitigating spillover risk. In parallel, public education and community awareness initiatives are essential to promote safer livestock handling and consumption practices. A key limitation in this study is the use of convenient sampling, which may affect the representativeness of the sampled population. Animals presented at livestock markets may differ from non-traded animals in age structure, health status, origin, or management practices, potentially introducing selection bias. Consequently, prevalence estimates should be interpreted with caution and may not be fully generalizable to the broader livestock population. However, livestock markets are critical nodes for animal movement and disease amplification, and market-based sampling remains epidemiologically relevant for assessing RVF exposure risk and informing surveillance efforts. Additionally, the limited number of animals sampled per visit may have introduced intra-market or intra-visit correlation; because clustering variables could not be defined, variance estimates were not adjusted for clustering, which may have resulted in overly precise confidence intervals.

## 5. Conclusions

This study reveals notable RVFV seroprevalence among camels and horses in northern Nigerian livestock markets, with clear species-specific and geographic variations in exposure. The results suggest that camels may play a role in maintaining RVFV in livestock populations, based on historical exposure indicated by IgG seropositivity. The epidemiological significance of transboundary livestock markets as potential sites for virus circulation is highlighted, emphasizing the value of serological surveillance in identifying populations with prior RVFV exposure. Integrating RVF surveillance into existing national and regional animal health frameworks is crucial for early detection, rapid response, and mitigation of possible zoonotic transmission.

## Figures and Tables

**Table 1:** Demographic and management characteristics of camels and horses in the study markets.

Variable	Level	Number	%
Species	Camel	812	72.7
	Horse	305	27.3
Location	Illela livestock market	230	20.6
	Maiduguri livestock market	257	23.0
	Maigatari livestock market	325	56.4
Age	Young	601	53.8
	Adult	516	46.2
Sex	Female	530	47.4
	Male	587	52.6

**Table 2:** RVFV Seroprevalence in camels and horses across livestock markets in Nigeria.

Species	No. Samples Tested	No. Positive Samples (%)	95% CI	*p*-Value
**Camel**				
*Ilela*	230	80 (34.8%)	28.7–41.4	*p* < 0.05
*Maiduguri*	257	52 (20.2%)	15.6–25.8	
*Maigatari*	325	66 (20.3%)	16.2–25.2	
Total	812	198 (24.4%)	21.6–27.4	*p* < 0.05
**Horse**				
*Ilela*	0	0 (0.0%)	-	
*Maiduguri*	0	0 (0.0%)	-	
*Maigatari*	305	12 (3.9%)	2.1–7.0	
Total	305	12 (3.9%)	2.1–7.0	
**Overall**	1117	210 (18.8%)	16.6–21.2	

**Table 3:** Risk factors for RVFV seropositive camels.

Characteristics	No. Samples Tested	No. Positive Samples (%)	95% CI	*p*-Value	Odds Ratio (95% CI)
**Age Category**	812				
Young	480	126 (26.2%)	22.4–30.4	0.137	1.29 (0.92–1.79)
Adult **^a^**	332	72 (21.7%)	17.4–26.5		1.00
**Sex**	812				
Male	419	96 (22.9%)	19.0–27.3	0.313	0.85 (0.62–1.17)
Female **^a^**	393	102 (26.0%)	21.7–30.6		1.00

**^a^** Reference category; odds ratios are estimated relative to the reference group (OR = 1.00).

**Table 4:** Risk factors for RVFV seropositive horses.

Characteristics	No. Samples Tested	No. Positive Samples (%)	95% CI	*p*-Value	Odds Ratio (95% CI)
**Age Category**	305				
Young	121	3 (2.5%)	0.5–7.1	0.293	2.02 (0.54–7.63)
Adult **^a^**	184	9 (4.9%)	2.3–9.1		1.00
**Sex**	305				
Male	168	3 (1.8%)	0.4–5.1	0.046	0.27 (0.07–0.97)
Female **^a^**	137	9 (6.6%)	3.2–12.5		1.00

**^a^** Reference category; odds ratios are estimated relative to the reference group (OR = 1.00).

## Data Availability

For reasons of confidentiality, the data used in this study are not published. Selected information may be shared with interested upon request and approval of the institute and herd owners.
